# Combined therapy guided by multimodal imaging of fifteen retinal capillary hemangioblastomas in a monocular Von Hippel- Lindau syndrome case report

**DOI:** 10.1186/s12886-022-02409-8

**Published:** 2022-05-06

**Authors:** Ju Guo, Liping Du, Pengyi Zhou, Xiaohong Guo, Fangfang Dai, Xuemin Jin

**Affiliations:** 1 Department of Ophthalmology, The First Affiliated Hospital of Zhengzhou University, Zhengzhou, 450000 China; 2 People’s Hospital of Zhengzhou University & Henan Eye Institute, Zhengzhou, 450000 China

**Keywords:** Multiple retinal capillary hemangioblastomas, Swept-source optical coherence tomography angiography, Von Hippel-Lindau syndrome, Precise combined therapy, Case report

## Abstract

**Background:**

To report the multimodal imaging and treatment of fifteen retinal capillary hemangioblastomas (RCHs) associated with Von Hippel-Lindau syndrome in a monocular patient during a long-term following-up, which supply high-resolution exquisite SS-OCTA images (VG200; SVision Imaging, Ltd., Luoyang, China) and management experience about multiple RCHs.

**Case presentation:**

A 34-year-old monocular male patient complained decreased visual acuity (20/100) without pain and redness in the left eye five years ago. Von Hippel-Lindau syndrome were diagnosed with genetic testing. He, his son and daughter all carried a heterozygosity missense variant c.499C > T (p. Arg167Trp) in the Hg19 gene, a VHL gene located in Chr3:10,191,506. Fifteen RCHs were identified by the application of multimodal imaging, which including fundus photo, fundus autofluorescence (FAF), B-scan ultrasonography (US), fluorescein fundus angiography (FFA), indocyanine green angiography (ICGA) and swept-source optical coherence tomography angiography (SS-OCTA). Transscleral cryotherapy and laser photocoagulation were performed to destroy the largest RCH with the size of 4 PD in diameter. Laser photocoagulation was conducted to seal the middle or tiny RCHs (< 1.5 PD) and their nourishing vessels. The retinal edema and exudative macular detachment were successfully relieved by intraocular injection of bevacizumab for 5 times. The RCHs in the left eye responded well to these treatments and best corrected visual acuity was 20/25 for three years. Three-month recall visits were recommended for him.

**Conclusion:**

For multiple retinal capillary hemangioblastomas in monocular patients, precise combined therapy guided by multimodal imaging has a profound impact on the management of new and recurrent RCHs.

## Background

VHL syndrome is an autosomal dominant inherited tumor syndrome caused by mutations of VHL gene, which is a tumour suppressor gene situated on chromosome 3p25 [1]. The mutations unbalance the expression of hypoxia-inducible factor, leading to tumours in the retina, central nervous system, kidneys and other organs [2]. Retinal capillary hemangioblastomas (RCHs) are benign tumours in nature and are always found in the periphery or juxtapapillary region. However, this syndrome usually leads to severe vision loss because of its serious complications, such as exudation, macular edema, retinal detachment, epiretinal membrane, and retinal and vitreous haemorrhage [3]. Therefore, it is necessary to timely detect and treat the RCHs in avoid of serious complications. In the past, the visualization of RCHs is mainly based on fluorescein fundus angiography (FFA), that can sketch the morphology of all VHL-related tumors in the retina. Swept-source optical coherence tomography angiography (SS-OCTA), a newly developed noninvasive ophthalmologic imaging technology, has been reported to provide a high-resolution representation for the true physical size of the RCHs [4, 5]. Nevertheless, the limited image range hamper the applications of SS-OCTA in clinical, so that it is important to get an accurate diagnosis by multimodal imaging of RCHs. Here, we report the multimodal imaging and treatment of fifteen retinal capillary hemangioblastomas (RCHs) associated with Von Hippel-Lindau syndrome in a monocular patient during the five-year following-up, which supply high-resolution exquisite SS-OCTA images and management experience about multiple RCHs. We obtained informed consent from the patient and his permission to report his case.

## Case presentation

**Fig. 1:**
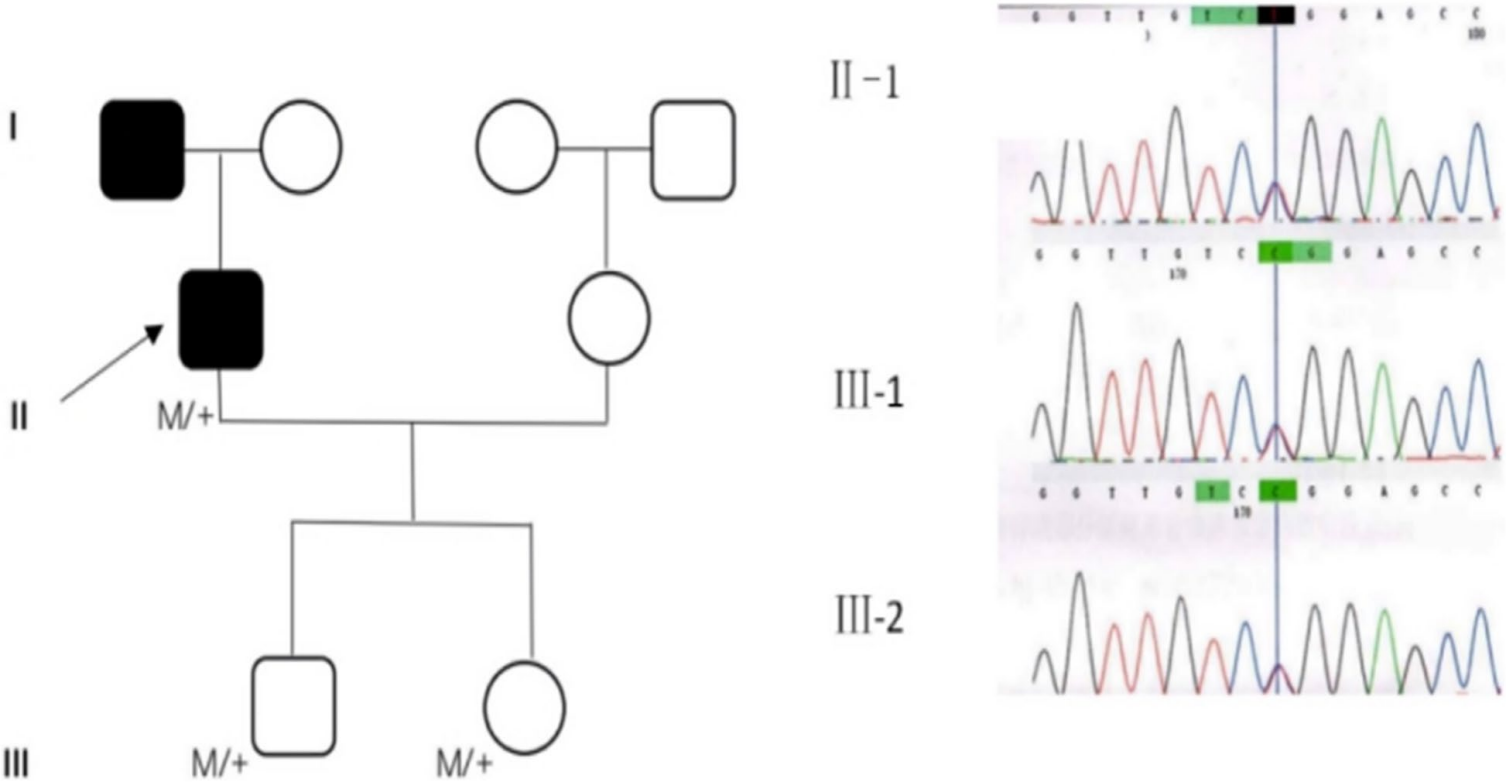
Pedigree chart and Sanger sequencing results of VHL gene variants using blood samples. The black square represents the patients who were clinically diagnosed with VHL. M/ + means heterozygous variants. The Sanger sequencing results show heterozygous missense variants in the VHL gene of patients II-1, III-1, and III-2

A 34-year-old male patient presented in our clinic on May 19, 2017, with a complaint of decreased visual acuity (20/100) in his left eye for several days. Laser photocoagulation had been performed at another hospital for the RCHs in his left eye half a year ago. He lost vision in his right eye after the surgical treatment of RCHs associated with VHL at the age of 30. The patient received multiple operations for cerebellar hemangioblastomas and adrenal pheochromocytoma when he was 25 years old. His father died from the complications of cerebellar hemangioblastomas associated with VHL. Genetic testing revealed that he, his son and daughter all carried a heterozygosity missense variant c.499C > T (p. Arg167Trp) in the Hg19 gene, a VHL gene located in Chr3:10,191,506 (Fig. 1).

**Fig. 2:**
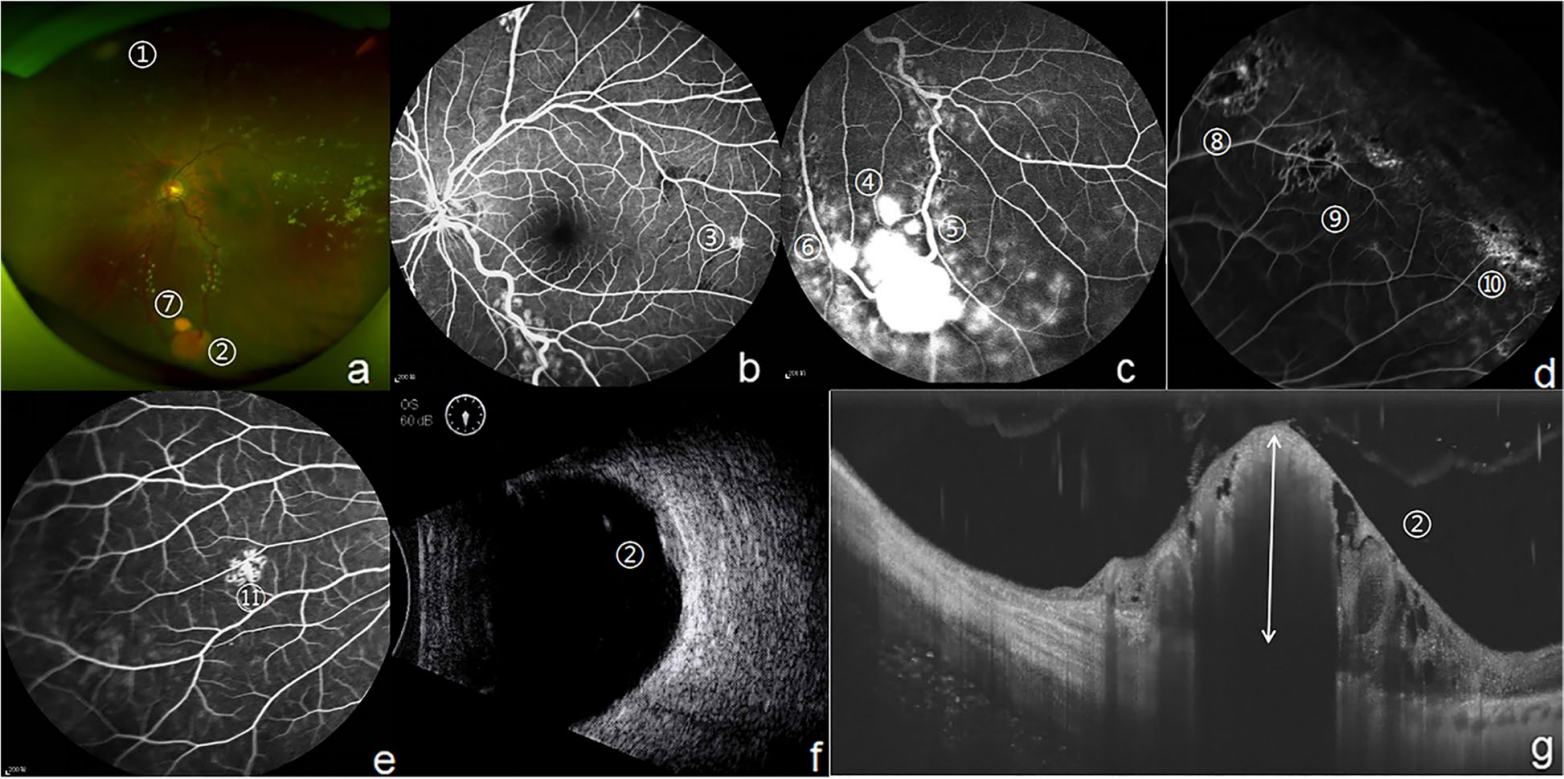
The first multimodal image evaluation of eleven RCHs in a Von Hippel-lindau syndrome patient on June 6^th^, 2017. **a**. Fundus photo identified huge RCHs (No.1, 2, 7 RCHs). **b**. FFA visualized a newly developed small RCH in temporal retina (No.3 RCH). **c**-**e**. FFA sketched the contours of several small peripheral RCHs (No.4–11 RCHs). **f**. B-scan ultrasonography displayed the largest oblate lesion with the size of 6 mm* 6 mm*2 mm was located in the inferior nasal retina (No.2 RCH). F. SS-OCT (single line scan mode) showed an elevated lesion wrapped much exudation and the largest RCH with the size of 2.15*2.60*1.35 mm, arrow represent the height (1.35 mm). (No.2 RCH). *RCH* retinal capillary hemangioblastomas, *FFA* fluorescein fundus angiography, *SS-OCT* swept-source optical coherence tomography

The fundus photo, B-scan ultrasonography (US), fluorescein fundus angiography (FFA) and SS-OCT (single line scan mode) visualized eleven RCHs in the left eye (Fig. 2). Fundus photo showed the huge RCHs along with diffuse exudation around their nourishing vessels (No.1, 2, 7 RCHs in Fig. 2). FFA distinguished the middle and tiny ones in retina (No.3–6, 8-11RCHs in Fig. 2). B-scan ultrasonography provided the size, shape and location of the largest RCH (No.2 RCHs in Fig. 2). SS-OCT measured the size of the largest RCH and visualized its full-thickness structure (No.2 RCHs in Fig. 2).

**Fig. 3:**
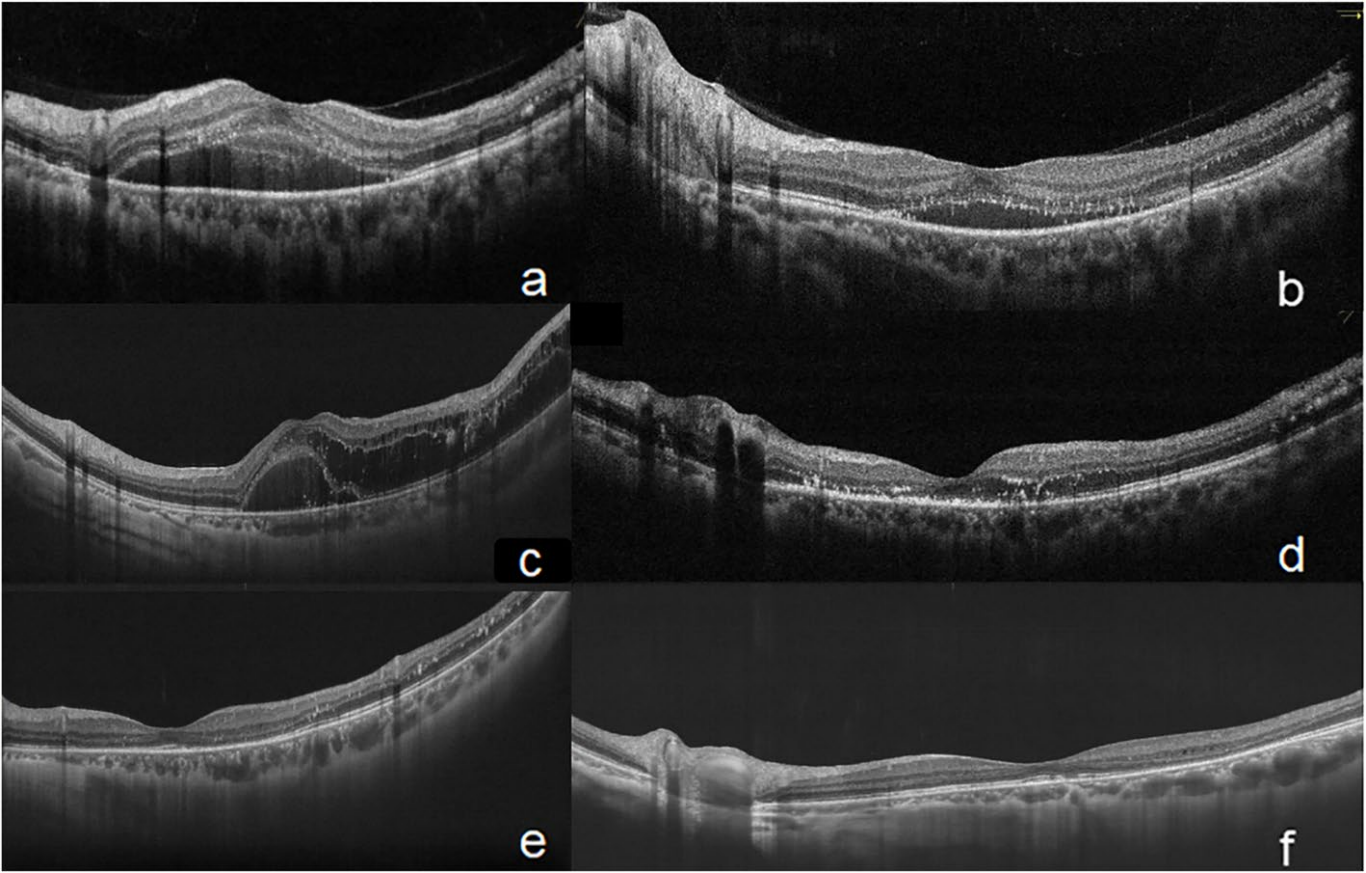
OCT (single line scan mode) image of exudation in the macular region during five-year following up. **a**.SD-OCT showed macular detachment with exudative subretinal fluid(SRF)at his first visit on May 19^th^, 2017. **b**. SD-OCT exhibited reduced SRF and exudation after twice anti-VEGF therapy and laser photocoagulation on September 10^th^, 2017. **c**. SS-OCT revealed recurrent exudative macular detachment complicated with macular cystoid edema on August 17^th^, 2018, one year after the combined therapy. **d**. SD-OCT visualized SRF and macular edema were completely absorbed with much exudation left after anti-VEGF therapy for four times on December 10^th^, 2018. **e**–**f**. Nine months and two years after the cryotherapy (August 10^th^, 2019, November 6^th^, 2020), the macular region was still in good structure by the observations of *SS-OCT* Spectral-domain optical coherence tomography, *SD-OCT* Swept- source optical coherence tomography, *SS-OCT* subretinal fluid, *SRF* subretinal fluid

**Fig. 4:**
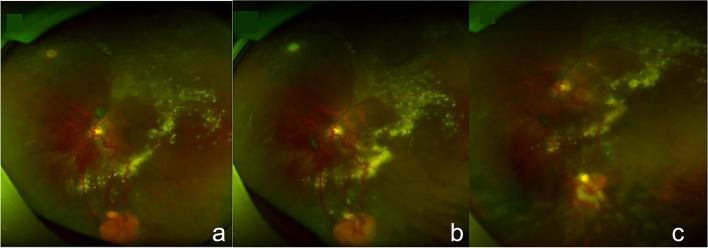
Fundus photo of rapidly enlarged RCHs and the therapeutic effect of cryotherapy. a. Fundus photo (taken in August 17^th^, 2018) showed an orange-red ellipse RCH with the diameter of approximately 3 PD was located in the nasal inferior retina, nourishing by two twisted and engorged vessels. b. Fundus photo (taken in December 10^th^, 2018) showed the RCH located in the nasal inferior retina was rapidly enlarged to 4 PD in diameter and its nourishing vessels dilated with much exudation. c. One month after cryotherapy, the RCH in the nasal inferior retina appeared to be pale and atrophied accompanied by reduced retinal exudation based on the fundus photo taken in January 28^th^, 2019. *RCH* retinal capillary hemangioblastomas, *PD* papillary diameter

After twice consecutive monthly intravitreal ranibizumab (0.5 mg) injections and followed by prompt laser photocoagulation (within a-week day after every injection), obviously absorption of subretinal fluid (SRF) and visual acuity improvement (20/25) were observed (Fig. 3a-b). One year later, the patient complained vision loss again with exudative macular detachment and enlarged RCHs in nasal inferior retina due to unregular reexamination after combined therapy (approximately 3PD in diameter) (Fig. 4a). Four monthly ranibizumab intravitreal injections were performed, the reduced transient SRF and improved vision were noted (Fig. 3c-d), but the RCHs still enlarged rapidly (approximately 4PD in diameter) (Fig. 4b).

**Fig. 5:**
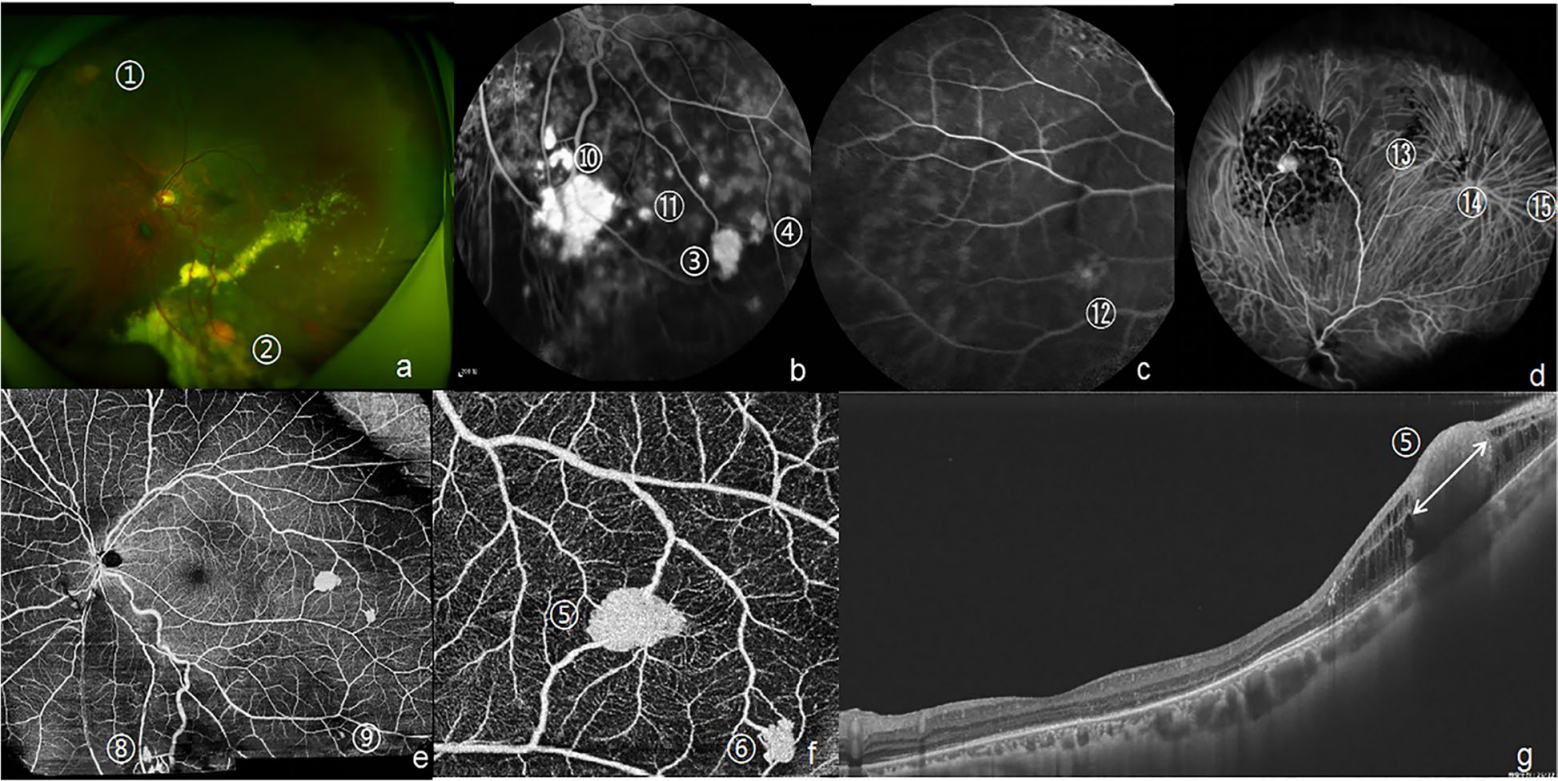
The second multimodal imaging of eleven RCHs in a Von Hippel-lindau syndrome patient on November 6^th^, 2020. **a**. Fundus photo showed a stationary white round-like RCH (1.5 PD in diameter) was in nasal superior retina (No.1 RCH) and an active orange round-like RCH (1 PD in diameter) along with diffuse exudation located in nasal inferior retina (No.2 RCH)**b**. FFA distinguished several middle RCHs from exudations (No.3–4, 10–11 RCH). c-d. FFA and ICGA showed four shrunk RCHs, which were small in previous evaluation, and now were almost traceless. e–f. SS-OCTA images at 15 × 12 mm^2^ and 6 × 6 mm^2^ sections with the center located on the fovea represented the angiography of the SCP, which respectively revealed a good structure in macular region and displayed small and tiny RCHs and their nourishing vessels clearly in posterior pole (No. 5–6, 8–9 RCH). g. SS-OCT (single line scan mode) showed a solid small hemangioma with the size of 1.35*0.84*0.62 mm, which was tiny in previous evaluation and overlooked in the laser treatment. (No.5 RCH). Arrow in figure g represent the length of No. 5 RCH, namely 1.35 mm. *RCH* retinal capillary hemangioblastomas, *FFA* fluorescein fundus angiography, *SS-OCT(*A) swept- source optical coherence tomography (angiography), *ICGA* indocyanine green angiography, *PD* papillary diameter, mm: millimeter, *SCP* superficial retinal capillary plexus (the layer between internal limiting membrane and 15 μm below inner plexiform layer)

**Fig. 6:**
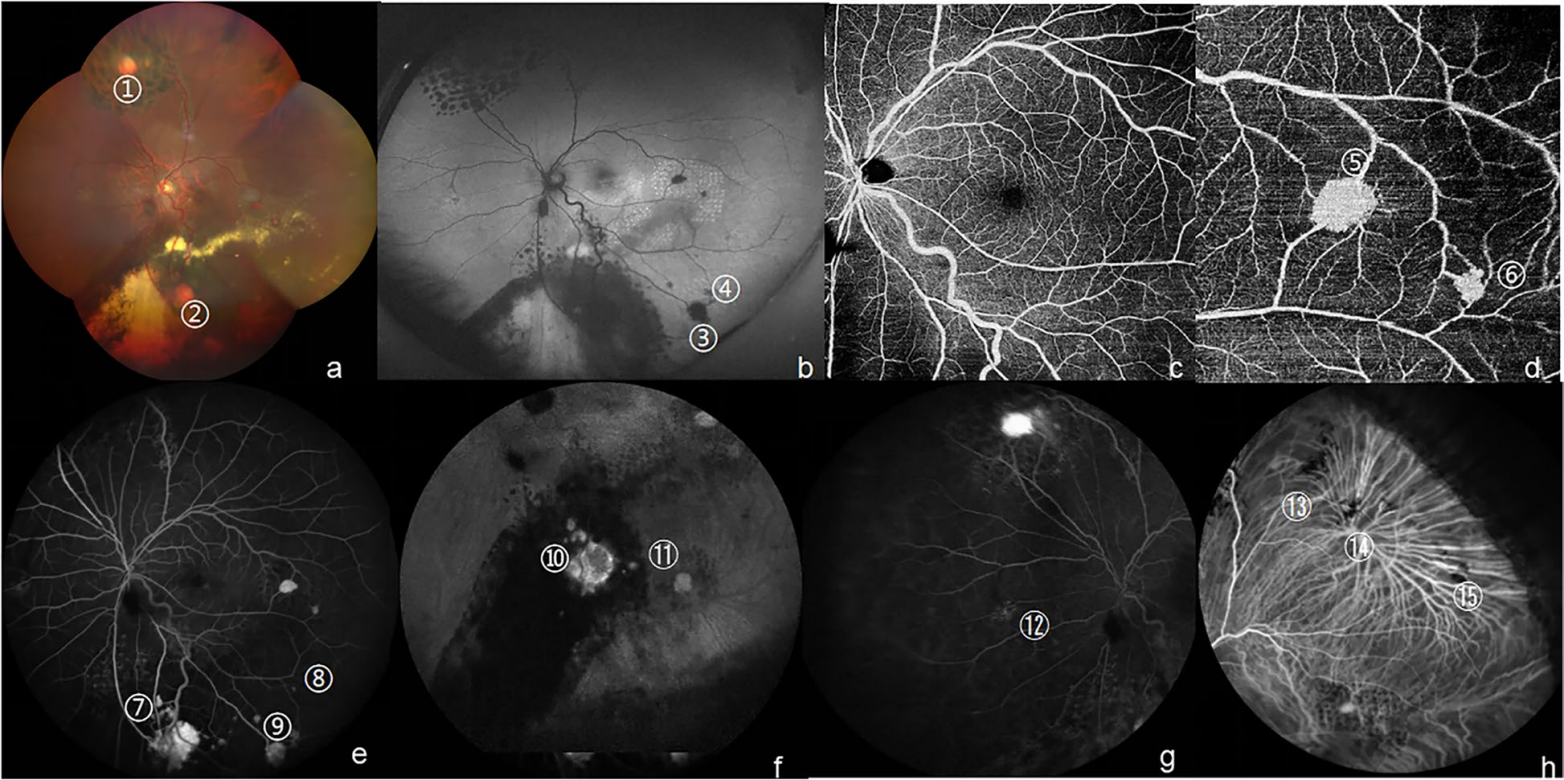
The third multimodal image evaluation of eleven RCHs in a Von Hippel-Lindau syndrome patient three month after the last laser treatment. **a**. Fundus photo identified huge RCHs and yellow-white exudation around RCHs (No.1–2 RCHs). **b**. FAF revealed the middle RCHs from dilated vessels (No.3 RCH). **c**-**d**. SS-OCTA images at 12 × 12 mm^2^ and 6 × 6 mm^2^ sections with the center located on the fovea showed the angiography of the SCP (**c**, **d**), which respectively showed the good structure in macular region and visualized No.5–6 RCHs begun to shrunk with decreased retinal blood flow density around the tumors after laser treatment. **e**. FFA visualized several small peripheral RCHs (No 7–9 RCHs). f. ICGA distinguished small RCHs from fluorescent leakage points (No.10–11). **g**-**h**. FFA and ICGA showed four traceless RCHs, which were atrophied by combined therapy. *RCH* retinal capillary hemangioblastomas, *FAF* fundus autofluorescence, *FFA* fluorescein fundus angiography, *ICGA* indocyanine green angiography, *SS-OCTA* swept- source optical coherence tomography angiography, *SCP* superficial retinal capillary plexus (the layer between internal limiting membrane and 15 μm below inner plexiform layer)

To control the enlarged RCHs and macular exudation, transscleral cryotherapy and intravitreal ranibizumab injection were performed under local anaesthesia in the left eye. One month after surgery, the largest RCH appeared to be pale and atrophied, accompanied by reduced exudation (Fig. 4c). Nine months after surgery, OCT images revealed that macular region remained a good structure and retinal exudation was reduced (Fig. 3e). Two years after surgery (November 6^th^, 2020), two newly developed RCHs temporal to macular were visualized by fundus examination using slit lamp due to the unregular reexamination. The multimodal imaging was recommended to this patient to find out all lesions in the retina in avoiding of serious complications. Fifteen RCHs were identified through detailed ophthalmological examination and multimodal imaging evaluation even if he had no complaint with vision loss (Fig. 5). The larger one with the size of 1.35*0.84*0.62 mm located in temporal to macular (No.5 RCH in Fig. 5) seemed to be exist in the first multimodal imaging evaluation, but it was so tiny that had been ignored during laser treatments. In addition, his irregular visit interrupted our observation and misled its diagnosis, so that No. 5 RCH grow rapidly during the two-year failure access. Then, three times continuous laser photocoagulations were performed around the RCHs and their nourishing vessels. Three months after laser therapy, the third multimodal image evaluation was conducted to observe the therapeutic effect and monitor active lesions (Fig. 6). The newly developed RCH (No.5 RCH in Fig. 5 and Fig. 6) responded well to laser treatments and best corrected visual acuity reaching 20/25. Following-up every three months were recommended for him to continuous monitor and treatment. The patient appreciated for the regained vision and maintained it for 3 years. Nevertheless, the financial burden of treatment may hinder his regular following-up.

## Discussion and conclusions

Recent reports found missense mutations were the most common while protein-truncating mutations have a higher probability for a severe course [6, 7]. In this case, the patient carried heterozygosity missense variant c.499C > T (p. Arg167Trp) in the Hg19 gene, but his severe course of this disease hints us that every patient, regardless of the kind of mutation should be screened very thoroughly. The treatment of RCHs associated with Von-Hippel Lindau syndrome differs due to the size, location, complications of the hemangiomas. In the earlier stage of RCHs, careful observation is recommended for small RCHs (less than 0.5 mm in diameter) without the complications of diffuse exudation [8]. It is usually effective for laser photocoagulation, photodynamic therapy (PDT), and external cryotherapy to treat small RCHs in peripheral retina (especially for RCH less than 1.5 mm in diameter) [8]. Brachytherapy or combined procedures have been recommended in the management of larger RCHs [9]. In cases with traction retinal detachment caused by RCHs, it is quite essential to treat by vitreoretinal surgery and tumour excision [10]. In addition, it has been demonstrated that the level of VEGF was elevated in the ocular fluids and in pathological specimens of eyes with VHL-associated RCH, so that the ophthalmologists have attempted to treat VHL-associated fundus lesions by the blockade of the VEGF signaling axis [11, 12]. Occasionally, intravitreal anti-VEGF therapy has been reported to be useful in the reduction of exudation and lipid deposition without significant regression for VHL-related RCHs in recent case reports [13–15]. In this case, the effect of intravitreal anti-VEGF therapy on the exudation and lipid deposition was consistent with previous reports.

In this report, the largest RCH (No.2 RCH)was orange-red appearance with the size of 4 PD in diameter and accompany by diffuse exudation, which should be managed by invasive treatments, namely brachytherapy, PDT, or tumour resection. However, these treatments may increase the incidence of macular edema and vision loss [16]. Therefore, cryotherapy was conducted to destroy the largest RCH by the consideration of rehabilitating vision in this monocular patient. Laser photocoagulation was performed to seal the middle or tiny RCHs (< 1.5 PD) and their nourishing vessels. The retinal edema and exudative macular detachment were successfully relieved by intraocular injection of bevacizumab for 5 times. Ultimately, the patient benefited a lot from the combined therapy and maintained a good vision for a long term.

SS-OCTA was a newly developed technology to visualize the microvasculature of the retina and choroid [17]. Previous research has demonstrated that SS-OCTA was advantage in detecting and measuring the size of RCHs in the posterior pole than other ophthalmological technologies [18]. It has also been elected as most suitable imaging technology to monitor the RCHs due to it can provide a high-resolution image of hemangiomas and assessment for their size [5]. In this case, SS-OCTA was applied to identify tiny RCHs and figure out the structure and size of RCHs in posterior pole. What’s more, SS-OCTA depicted exquisite images of two RCHs in Fig. 5f. It contributed greatly in the long term following-up. FFA shows its advantage at sketching the contours of RCHs in peripheral retina, but can’t give a true assessment for their shape and texture features. Thus, it had been used to find out all potential RCHs in periphery in thrice multimodal image evaluations. What’s more, ICGA distinguished small RCHs in periphery from redundant fluorescent leakage points, due to its high protein binding rate. FAF can recognize the hypo-fluorescence middle sized RCHs from dilated vessels. B-scan ultrasonography give the information about the location and size of largest RCH, which rose above the retinal surface. In this case, all common ophthalmological imaging technologies were used to evaluate these fifteen RCHs, and their advantages were further discussed, which may have a profound impact on early detection of new and recurrent RCHs. However, the largest limitation of this case was the incident in the process of therapy due to his unregular reexamination.

In conclusion, it is safe and beneficial to utilize the combined therapy, namely transscleral cryotherapy, intravitreal ranibizumab injection and laser photocoagulation, guided by the multimodal imaging evaluation to treat multiple RCHs associated with Von-Hippel Lindau syndrome. In future following-up, it is important to apply the two magic weapons (multimodal imaging and combined therapy) to manage the RCHs.

## Data Availability

All data generated or analyzed during this study are included in this published article. The whole genome sequence result was provided in the supplementary file.

## Abbreviations

VHL: Von Hippel-Lindau
RCH: retinal capillary hemangioma
VEGF: vascular endothelial growth factor
FAF: Fundus autofluorescence
FFA: Fluorescein fundus angiography
ICGA: Indocyanine green angiography
SS-OCTA: Swept-source optical coherence tomography angiography
